# Supplementary material for: Learning to detect AI texts and learning the limits
Source: PLoS One. 2025 Oct 15;20(10):e0333007. doi: 10.1371/journal.pone.0333007 (PMC12527182; doi:10.1371/journal.pone.0333007)
Supplement: S2 File — All data and scripts used in the study, some additional descriptive statistics, scalable formate figures. (ZIP) [file pone.0333007.s002.tgz › data and scripts/OSF/www/index.htm]

LLM Text Evaluation


# Experiment na rozpoznávání textů generovaných umělou inteligencí

Právě se účastníte vědeckého experimentu o poznávání textů psaných umělou inteligencí. Vaše anonymizované odpovědi budou zaznamenány pro vědecké účely. Z experimentu můžete kdykoliv odstoupit tím, že experiment nedokončíte a zavřete. V takovém případě budou vaše odpovědi vymazány. Experiment by vám měl zabrat přibližně 25 minut. Nejprve vás požádáme o vyplnění krátkého demografického dotazníku a pak bude následovat přesná instrukce.

Prosíme, abyste experiment vyplnili na počítači, ne na mobilním telefonu.

V případě dotazů či problémů při vyplňování experimentu se obraťte na Jiřího Miličku (experiment@milicka.cz).

Pokračovat

# Informovaný souhlas

Prosíme, přečtěte si Informovaný souhlas s účastí ve výzkumu a kliknutím na políčko umístěné dole potvrďte, že jste dokument četl/a a souhlasíte s ním.

Informovaný souhlas s účastí na studii.

Četl/a jsem Informovaný souhlas a souhlasím s ním.

Pokračovat

## Demografický dotazník

V následující části bychom vás chtěli poprosit o vyplnění otázek týkajících se demografických údajů a vašich postojů vůči umělé inteligenci.

Pohlaví:

Muž
 Žena
 Jiné
 Nechci uvést

Věk:

Mateřský/é jazyk/y:

Čeština

Čeština a jiný/é: 

Čeština není můj mateřský jazyk, tím je:

Vyskytují se u vás poruchy čtení či porozumění textu (např. dyslexie)?

Ano
 Ne
 Nevím

Studujete v současné době?

Ano
 Ne

Pokud studujete, uveďte, co právě studujete:  
V případě více studií zvolte studium vyššího stupně

Základní škola
 Střední škola (s maturitou i bez)
 Vyšší odborná škola
 Bakalářské studium
 Magisterské studium
 Doktorské studium
 Jiné

Jaké je zaměření vašeho studia? (více možností)

Filologické (bohemistika, lingvistika, překladatelství, učitelství jazyka, literární věda...)
 Ostatní humanitní a společenskovědné (historie, psychologie, sociologie, ekonomie, umělecké obory, právo, filosofie...)
 Informatika, strojové učení
 Ostatní přírodovědné a technické (biologie, chemie, fyzika, zdravotnické obory...)
 Jiné/Nelze zařadit

Pokud nestudujete, uveďte vaše nejvyšší dokončené vzdělání:

Bez vzdělání
 Základní
 Střední (s maturitou i bez)
 Vyšší odborné
 Vysokoškolské – Bakalářské
 Vysokoškolské – Magisterské
 Vysokoškolské – Doktorské
 Jiné

Jaké bylo zaměření vašeho studia?

Filologické (bohemistika, lingvistika, překladatelství, učitelství jazyka, literární věda...)
 Ostatní humanitní a společenskovědné (historie, psychologie, sociologie, ekonomie, umělecké obory, právo, filosofie...)
 Informatika, strojové učení
 Ostatní přírodovědné a technické (biologie, chemie, fyzika, zdravotnické obory...)
 Jiné/Nelze zařadit

Další strana

Jak často pracujete s velkými jazykovými modely (např. ChatGPT, Claude, Gemini apod.)?

Každý den
 Několikrát týdně (více než jednou týdně, ale nikoli denně)
 Zhruba jednou týdně
 Několikrát měsíčně (ale ne každý týden)
 Přibližně jednou měsíčně
 Méně často než jednou měsíčně
 Nikdy
 Nevím/Nedokážu odpovědět

Do jaké míry souhlasíte s následujícími výroky?

Umělá inteligence může zlepšit kvalitu našeho každodenního života.

Zcela nesouhlasím

Zcela souhlasím

Umělá inteligence představuje riziko pro lidskou bezpečnost.

Zcela nesouhlasím

Zcela souhlasím

Automaticky generované texty mohou být stejně kvalitní jako texty napsané lidmi.

Zcela nesouhlasím

Zcela souhlasím

Automaticky generované texty postrádají lidskou kreativitu a osobitý styl.

Zcela nesouhlasím

Zcela souhlasím

Umělá inteligence není schopná opravdového porozumění, jako to umí člověk.

Zcela nesouhlasím

Zcela souhlasím

Umělá inteligence produkuje gramaticky správné texty.

Zcela nesouhlasím

Zcela souhlasím

Raději se vyhýbám užívání umělé inteligence při psaní textů, na kterých mi záleží.

Zcela nesouhlasím

Zcela souhlasím

Mám obavy, že lidé kvůli užívání umělé inteligence hloupnou.

Zcela nesouhlasím

Zcela souhlasím

Fascinuje mě, co vše umělá inteligence při psaní textů dokáže.

Zcela nesouhlasím

Zcela souhlasím

Pokračovat

## Instrukce

Pokračovat

Zde uvidíte dva úryvky textu. Jeden je psaný člověkem, druhý generovaný umělou inteligencí.

Text 1

Byl pozdní večer - první máj -
  
 večerní máj - byl lásky čas,
  
 hrdliččin zval ku lásce hlas,
  
 kde borový zaváněl háj.

Text 2

Večerní mlhou tichý šum,
  
 algoritmus lásky plní dům,
  
 data se mísí v digitální tmě,
  
 romantika kódů zní v každé větě.

Až si texty přečtete, nejprve odpovězte na subjektivní otázku o tom, který text se vám čte lépe. Tato otázka nemá správnou odpověď, jde o vaši osobní preferenci. Prosím, vyberte si jednu z možností, i když vám může připadat těžké se rozhodnout.

Který text se vám lépe čte?

Text 1

Text 2

V druhé otázce odpovíte na to, který text podle vás napsal člověk. Existuje správná odpověď.

Který text je napsaný člověkem?

Text 1

Text 2

V poslední otázce označíte, nakolik jistí jste si svou odpovědí o tom, který text napsal člověk.

Nakolik jste si svou odpovědí jisti?

Velmi nejistý/á

Velmi jistý/á

Kliknutím na tlačítko OK se vaše odpověď uloží a už se k ní nebudete moci vrátit. Také vám ukážeme, zda jste odpověděli správně či ne.

OK

### Nyní bude následovat samotný experiment.

OK

### Task 1 out of X

### Text 1

### Text 2

Který text se vám lépe čte?

Text 1

Text 2

Který text je napsaný člověkem?

Text 1

Text 2

Nakolik jste si svou odpovědí jisti?

Velmi nejistý/á

Velmi jistý/á

OK

Pokračovat

## Kontrolní otázka

O čem byly texty, které jste zrovna četl/a?

Pokračovat

## Děkujeme Vám za účast na experimentu.

Všechny úlohy jsou dokončeny. Prosím napište nám sem o svých strategiích při plnění úkolů, popřípadě cokoli dalšího, co se týká tohoto experimentu. Pak můžete kliknout na tlačítko a dozvědět se správné odpovědi a celkový výsledek.

Odeslat odpovědi a ukázat výsledky

Poslat e-mailovou adresu (kvůli zápočtu v LABELS)

## Výsledky byly úspěšně odeslány a experiment je u konce...

Pokud potřebujete splnit účast v experimentu v rámci LABELS, uveďte e-mail (stejný jako v registračním systému). Pokud experiment neplníte v rámci žádného předmětu, napište pouze X (nebo nějakou zprávu tvůrcům experimentu:-). E-mail nebude (a ani technicky nemůže být) nijak spojen s vašimi odpověďmi.

Odešli e-mail  
Konec

E-mail byl odeslán.
